# Supplementary material for: Regulatory feedback between VEGF and ERK pathways controls tip-cell expression during sea urchin skeletogenesis
Source: Development. 2025 Jul 15;152(13):dev204684. doi: 10.1242/dev.204684 (PMC12315553; doi:10.1242/dev.204684)
Supplement: Supplementary information [file develop-152-204684-s1.pdf]

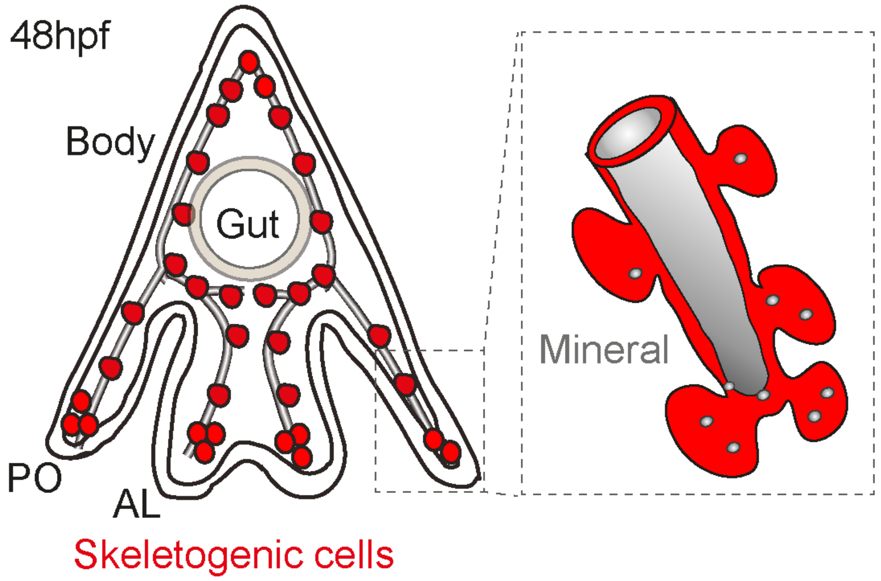

Fig. S1. Schematic diagram of sea urchin larval skeletal elongation at 48hpf. The skeletogenic cells are marked in red and the mineral in gray. The skeletogenic cells are marked in red and the mineral in gray. At 48hpf the sea urchin pluteus has formed its body, post-oral (PO) and antero-lateral (AL) rods. Enlargement shows the spicule tubular cavity and the skeletogenic cells that share it. The spicule grows by the deposition of vesicles bearing mineral ions and matrix proteins into the inner membrane engulfing the biomineral at the tips of the rods.

Development • Supplementary information

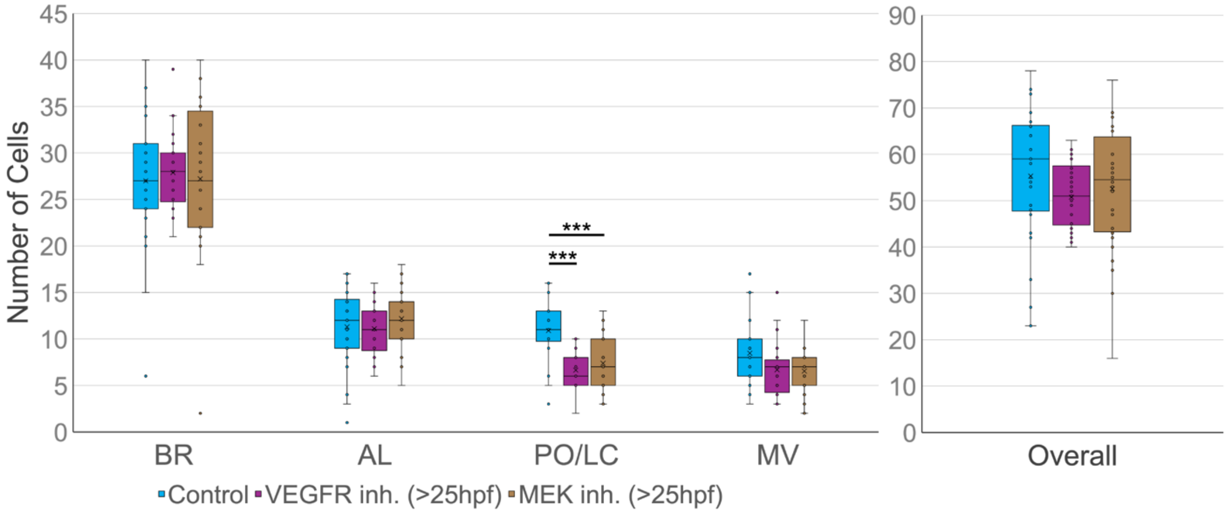

Fig. S2. VEGF and MEK inhibition influence the number of cells along the post-oral rods. A boxplot depicting the number of cells counted in each skeletal rod, body (BR), antero-lateral (AL), post-oral/lateral clusters (PO/CL), and mid-ventral (MV) and overall. Blue indicates control embryos, purple indicates VEGFR-inhibited embryos (150nM Axitinib >25hpf), and brown indicates MEK-inhibited embryos (10µM U0126 >25hpf). Statistical significance was measured using one-way ANOVA tests along with bonefferi post-hoc tests when possible. If needed, Kruskal-Wallis tests (non-parametric one-way ANOVA) along with pairwise tests between groups were performed. p<0.001 for both significant results. Experiments were conducted in three independent biological replicates.

Development • Supplementary information

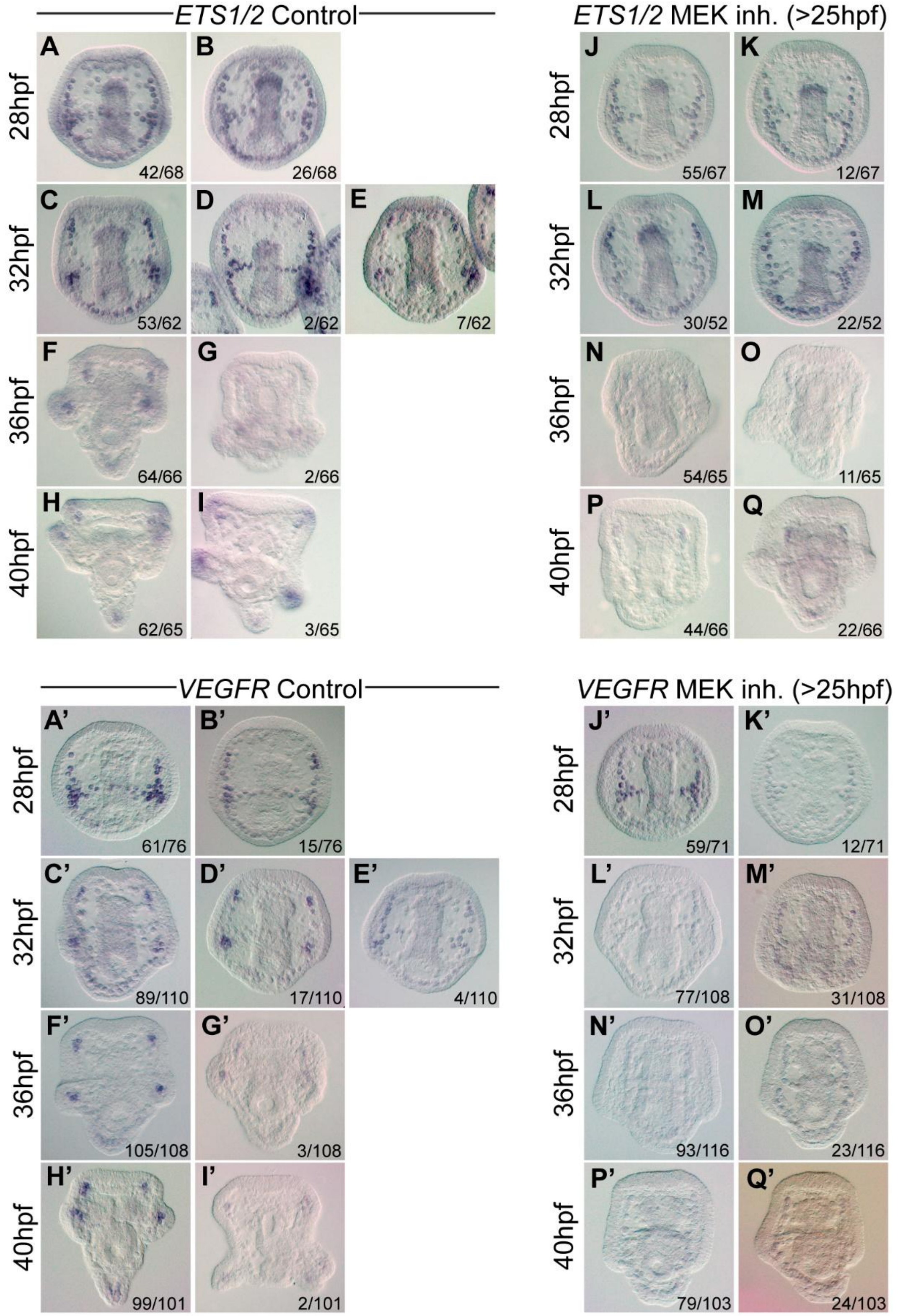

Fig. S3. ERK signaling is required for normal expression of *ets1/2 and* VEGFR, complete set of expression patterns. Representative images showing the spatial expression of genes *ets1/2* (A-Q) and *VEGFR* (A'-Q') under control (A-I, A'-I'), and MEK-inhibition (10µM U0126 >25hpf, J-Q, J'=Q') conditions. Numbers on the bottom right of each panel indicate the number of embryos seen with the spatial expression pattern shown in the image. Experiments were conducted in two (*ets1/2*, *VEGFR* at 28hpf) or three (*VEGFR* at 32, 36 and 40hpf) independent biological replicates.

Development • Supplementary information

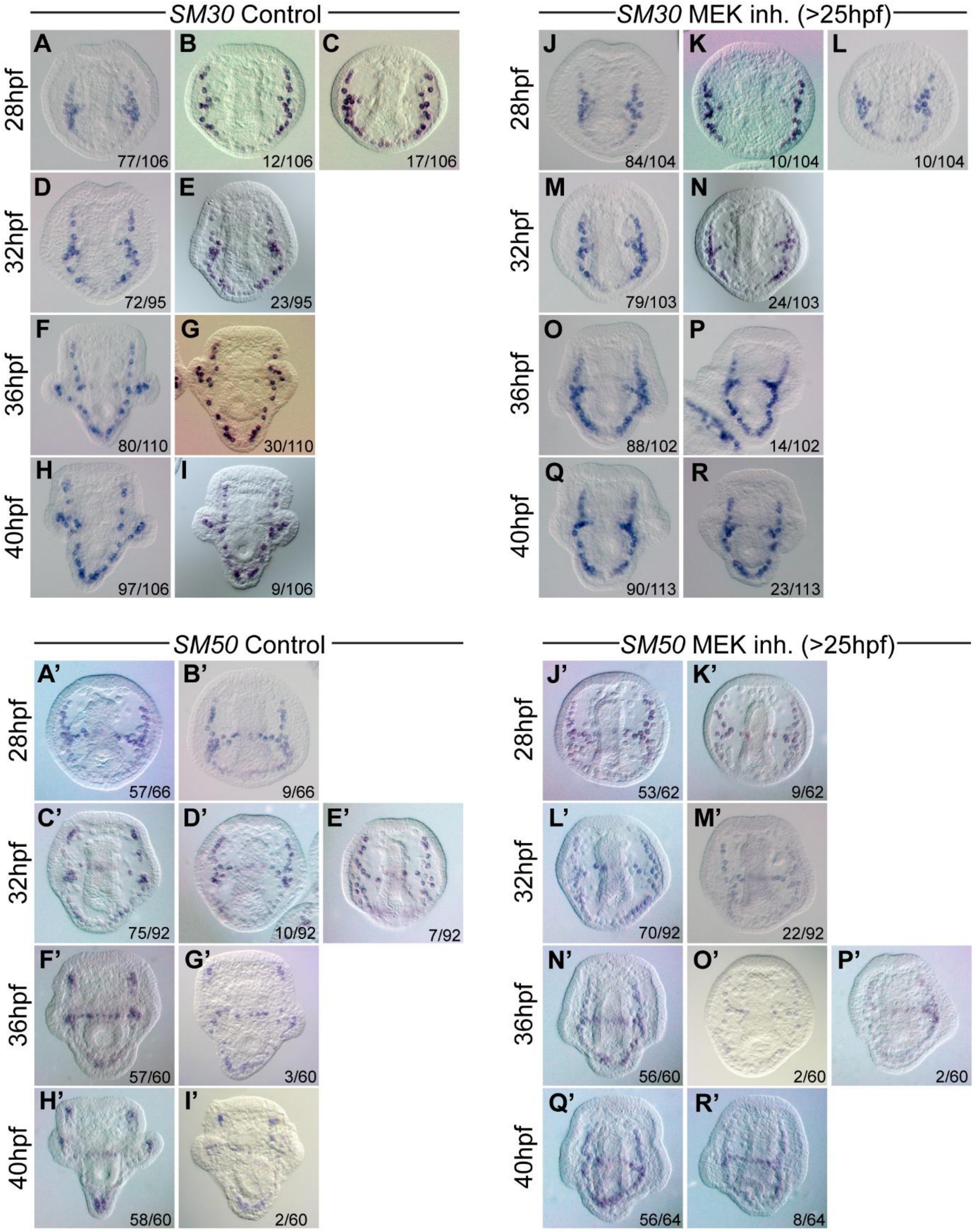

Fig. S4. ERK signaling is required for normal expression of *SM50* but not *SM30*, complete set of expression patterns. Representative images showing the spatial expression of genes *SM30* (A-R) and *SM50* (A’-R’) under control (A-I, A’-I’), and MEK-inhibition (10µM U0126 >25hpf, J-R, J’=R’) conditions. Numbers on the bottom right of each panel indicate the number of embryos seen with the spatial expression pattern shown in the image. Experiments were conducted in two (SM50 at 28, 36, 40hpf) or three (SM50 at 32hpf, SM30) independent biological replicates.

Development • Supplementary information

Table S1. Primer sequences used for qPCR experiments

Gene name	Primer ID	Sequence 5' 3'
Alx1	Forward primer for Alx1	AATTTGCAGAACCTCCGACG
	Reverse primer for Alx1	TCATTGAGGAGTGGTTGGCT
ERG	Forward primer for ERG	CTACACCTGAGGAGCGATCG
	Reverse primer for ERG	ACCCACTGCTGTACATGCTC
Ets1/2	Forward primer for Ets1/2	CCAACATGCATCTGGACGAG
	Reverse primer for Ets1/2	CCGGACCGCCAATCATTATG
Notch	Forward primer for Notch	GATGTCAAAGTAGCAACGGGCTTGA
	Reverse primer for Notch	CGTCTGGCAGTTCTTACCATAGAAGC
Pitx1	Forward primer for Pitx1	ACAAGTTCGCCGTAACTTCG
	Reverse primer for Pitx1	GAGGGTGTTGTCGCTTATCC
SM30	Forward primer for SM30	AGGTGGTTTCCCTGGACAAG
	Reverse primer for SM30	TTGGGCATGTCTCCTGTCTT
SM50	Forward primer for SM50	GGACCTGCTAAAATGGGCTC
	Reverse primer for SM50	TCCATCCAAGCCAGATCTCA
VEGFR	Forward primer for VEGFR	CACTGGGAGATATCGGTGCT
	Reverse primer for VEGFR	ACGGTTGCCCACGATAAATA
VEGF	Forward primer for VEGF	GCTCATGGTTCTCTCGAAGG
	Reverse primer for VEGF	CCCGCTGAGATAACATTGGT
Dlx	Forward primer for Dlx	GGGCATCCTCCAATTTATGA
	Reverse primer for Dlx	GCAAGGTATTGGGTCTGGTG

Ese	Forward primer for Ese	AGGATTGTGAGGCCCGTATT
Ese	Reverse primer for Ese	GGAACTCTACCGTCAAACCG
FoxA	Forward primer for FoxA	CAGGTATGGGAAGCATGGGA
	Reverse primer for FoxA	GCGTATCTCATCGACATGGC
FoxQ2	Forward primer for FoxQ2	CCACCATCAACCAAGAAACC
	Reverse primer for FoxQ2	TCCAGCTTCTTTCGTTGTCC
GCM	Forward primer for GCM	ATTTTCGACATCCCTGCTGC
	Reverse primer for GCM	CTTGAAGTACCTGCGGCATC
Nodal	Forward primer for Nodal	GCATTGAACTCCGCTCCAAA
	Reverse primer for Nodal	TCGCCGTCCTCACTAGAATC
SoxC	Forward primer for SoxC	GCAACAAATTCCAAACGGCC
	Reverse primer for SoxC	CATAGGTCGCTTGATGTGGC
Ubiquitin	Forward primer for Ubiquitin	CAAGACAAAGAAGGTATCCC
	Reverse primer for Ubiquitin	CTTGACGAAGATCTGCATAC

Table S2. Probe sequences used for WMISH experiments

**Gene name**	**Primer ID**	**Sequence 5' 3'**
SM50	Gifted to lab from Jeni Croce	
SM30	Forward primer for SM30	CCTCCCCCTTTCGTGTTATAAATG
	Reverse primer for SM30	GCAAAACAACTTCTTCGTCGGTC
VEGFR	Forward primer for VEGFR	GTTTTCACGTGCACCTTTCA
	Reverse primer for VEGFR	CGGTATCGGCCACACTGACG
Ets1/2	Forward primer for Ets1/2	CGACCTTAGTAACAAGAGCCGC
	Reverse primer for Ets1/2	CGGCTTTATCCTCTGTCAAATACTC
